# Reconstitution of TGFBR2-Mediated Signaling Causes Upregulation of GDF-15 in HCT116 Colorectal Cancer Cells

**DOI:** 10.1371/journal.pone.0131506

**Published:** 2015-06-26

**Authors:** Jennifer Lee, Fabia Fricke, Uwe Warnken, Martina Schnölzer, Jürgen Kopitz, Johannes Gebert

**Affiliations:** 1 Department of Applied Tumor Biology, Institute of Pathology, University Hospital Heidelberg, Heidelberg, Germany; 2 Cancer Early Detection, German Cancer Research Center (DKFZ), Heidelberg, Germany; 3 Functional Proteome Analysis, German Cancer Research Center (DKFZ), Heidelberg, Germany; University of Texas Health Science Center, UNITED STATES

## Abstract

Although inactivating frameshift mutations in the *Transforming growth factor beta receptor type 2* (*TGFBR2*) gene are considered as drivers of microsatellite unstable (MSI) colorectal tumorigenesis, consequential alterations of the downstream target proteome are not resolved completely. Applying a click-it chemistry protein labeling approach combined with mass spectrometry in a MSI colorectal cancer model cell line, we identified 21 *de novo* synthesized proteins differentially expressed upon reconstituted TGFBR2 expression. One candidate gene, the TGF-ß family member Growth differentiation factor-15 (GDF-15), exhibited TGFBR2-dependent transcriptional upregulation causing increased intracellular and extracellular protein levels. As a new TGFBR2 target gene it may provide a link between the TGF-ß branch and the BMP/GDF branch of SMAD-mediated signaling.

## Introduction

Colorectal cancer (CRC) can arise through three major pathways, characterized by chromosomal instability [1], CpG island hyper-methylation phenotype [2] and mismatch repair (MMR)-deficiency [3, 4]. Tumors that have lost normal MMR function display a high level of microsatellite instability (MSI) usually recognized as insertion/deletion mutations at short mononucleotide repeats (microsatellites) located in coding and non-coding gene regions. More than 90% of MSI colorectal cancers have acquired somatic insertion/deletion mutations in the coding mononucleotide repeat (A10) in exon 3 of the *Transforming growth factor beta receptor type 2* (*TGFBR2*) gene leading to translational frameshifts and impaired receptor signaling [5, 6]. The TGFBR2 protein is a serine-threonine kinase which, upon binding of its ligand TGF-ß1, confers canonical SMAD-mediated signaling [7]. TGF-ß signaling plays a key role in normal function of colon epithelial cells but its role in tumorigenesis seems to be controversial [8, 9]: in the early stages of tumor development, TGF-ß acts as a classical tumor suppressor [10], whereas it promotes EMT, migration and metastasis at later stages of tumor progression [11]. These apparently paradoxical biological features are determined by a large number of target proteins whose expression is regulated in a TGF-ß-dependent manner [12].

Most of these *bona fide* downstream targets of TGF-ß signaling have been identified by screenings at the transcriptional level whereas studies focusing on TGF-ß-dependent proteomic changes in colorectal tumor cells are rather limited and proteomic expression profiles cannot always be derived from transcriptome patterns. Although the TGF-ß signaling pathway has already been extensively characterized, its impact on protein expression dynamics that contribute to the biochemical/proteomic phenotype of colon cancer cells is not yet fully explored.

Since tumor cells in general and MSI tumor cells in particular have acquired a limited number of driver mutations that contribute to tumor development among a large number of irrelevant passenger mutations, delineation of complex proteomic changes with specific driver mutations is a major challenge. In order to understand how malfunction of a single member of the TGF-ß signaling pathway, the TGFBR2, alters the cellular proteomic and glycomic landscape in MSI colorectal cancer cells, we previously established a MSI cell line model system that enables doxycycline (Dox)-regulated reconstitution of TGFBR2 expression and signal transduction in an isogenic background [13].

Using this HCT116-TGFBR2 model cell line in combination with a click chemistry approach for specific isolation of the *de novo* proteome [14] and subsequent mass spectrometry (MS) analysis, we identified a limited number of proteins that turned out to be newly expressed or whose synthesis was abolished upon TGFBR2 expression. One of these candidate targets, the Growth and differentiation factor-15 (GDF-15) showed a TGFBR2-dependent transcriptional upregulation that correlated with increased levels of intracellular and secreted protein. These data suggest that GDF-15 is a TGF-ß-responsive gene.

## Materials and Methods

### Cell culture

Cell lines were grown in RPMI 1640 medium (Life Technologies, Karlsruhe, Germany) supplemented with 10% FBS (Life Technologies, Karlsruhe, Germany), 100 U/ml penicillin and 100 μg/ml streptomycin (Life Technologies, Karlsruhe, Germany) using standard conditions. The generation of the doxycycline-inducible HCT116-TGFBR2 cell clones was reported previously [13]. Briefly, the wildtype *TGFBR2* gene was integrated into the colorectal cancer cell line HCT116, which harbors biallelic inactivating *TGFBR2* frameshift mutations, using recombinase-mediated cassette exchange (RMCE) resulting in two independent cell clones, #5 and #22, with different single copy integration site.

### Metabolic Labeling

For metabolic labeling experiments, 4x10^6^ cells were seeded in triplicate on 10 cm plates. Next day, medium was replaced and cells were either grown in absence or presence of 0.5 μg/ml doxycycline (Sigma, Taufkirchen Germany). After 11 h, cells were washed with PBS (Life Technologies, Karlsruhe, Germany), grown for 30 min in methionine-free RPMI 1640 medium (Sigma, Taufkirchen Germany) and subsequently incubated with 10 ng/ml TGF-ß1 (Cell Signaling, Danvers, USA) and the labeling reagent (40 μM L-Azidohomoalanine (AHA) or 4.625 MBq [^35^S]-L-methionine) in presence or absence of Dox for 4 h (protein lysate) or 24 h (secreted proteins). As a control for identifying only AHA-labeled newly synthesized proteins, cells were grown in triplicate in absence of AHA and in presence of 200 μg/ml cycloheximide (CHX), an inhibitor of protein biosynthesis. For analyzing the effect of GDF-15 on pSmad2 signaling, 100 ng/ml human recombinant GDF-15 (G3046; Sigma, Taufkirchen, Germany) was added to the cells for 1 h. Cells were washed 3x with PBS, harvested and centrifuged at least 10 min at 1000 g at 4°C in PBS containing 1x protease inhibitor cocktail (Roche, Mannheim, Germany). Cell pellets were directly resuspended in lysis buffer. Medium of the radioactive-labeled cells was collected after 24 h, centrifuged at 1000 g at 4°C for least 10 min and the supernatant was subjected to GDF-15 immunoprecipitation.

### Click-iT Reaction

After metabolic AHA labeling (C10102; Life Technologies, Karlsruhe, Germany), cell pellets were lysed with 100 μl of 1% SDS in 50 mM Tris-HCl, pH 8, supplemented with protease inhibitor cocktail, sonicated for 30 s and incubated at least 30 min at 4°C while rotating as described previously [14]. After centrifugation at 4°C for 30 min at 12,000 g, protein concentration was determined by Bradford Assay (Bio-Rad Laboratories GmbH, Munich, Germany). 200 μg of protein was used to react with 40 μM Biotin Alkyne in DMSO (B10185; Life Technologies, Karlsruhe, Germany) according to the manufacturer’s instructions. After methanol/chloroform (4:1) precipitation, the precipitates were resolubilized in 200 μl RIPA buffer, sonicated for 30 s and rotated overnight at 4°C. To remove unsolubilized material, the protein sample was centrifuged at 4°C for 20 min at 12,000 g. The supernatant was then incubated on a rotator with 80 μl slurry of streptavidin-coated magnetic beads (Dynabeads MyOne Streptavidin T1, Life Technologies, Karlsruhe, Germany), that had been washed 3x with PBST (PBS/0.01% Tween 20). After 2 h at 4°C, beads were washed 3x with 1 ml PBST containing 2% SDS followed by final washes with PBS and 40 mM ammonium bicarbonate.

### Mass Spectrometric Analysis

For mass spectrometry, samples were prepared and analyzed as described previously [14]. Briefly, protein-loaded beads were reduced, alkylated and trypsinysed at 37°C overnight. After addition of TFA the sample was analyzed by nanoLC ESI-MS/MS on LTQ Orbitrap XL mass spectrometer (Thermo Scientific, Karlsruhe, Germany). The mgf-files generated by the Xcalibur software (Thermo Scientific, Karlsruhe, Germany) were used for database searches with the MASCOT search engine (Matrix Science; version 2.4) against SwissProt database (SwissProt version 2013_02 (539165 sequences; 191456931 residues)) with the taxonomy set to human. Candidate proteins were classified as differentially expressed (TGFBR2-deficient or-proficient) if detected in at least one of three biological replicates in each of both cell clones (#5 and #22) but not in parental HCT116-Tet-On cells.

### Quantitative Real Time (qRT)-PCR

1 μg of total RNA was isolated using the RNeasy Kit (Qiagen, Hilden, Germany) and reverse transcribed with Oligo-dT primers and SuperScript II reverse transcriptase according to the manufacturer‘s protocol (Life Technologies, Karlsruhe, Germany). Real-time PCR analysis was performed using PowerSYBR Green Master Mix (Applied Biosystems, Darmstadt, Germany) and specific primers for TGFBR2 (for: 5´-CGGCTCCCTAAACACTACCAA-3´, rev: 5´-AACAAATTGGACTAATCCGGA-3´) and GDF-15 (for: 5´-AAGATTCGAACACCGACCTC-3´, 5´-AGAGATACGCAGGTGCAGGT-3´). Triplicates of different cDNA samples (-/+ Dox) were analyzed in the StepOnePlus thermo-cycler (Applied Biosystems, Darmstadt, Germany) using the following program: 95°C for 10 min, followed by 40 cycles of 95°C for 15 s and 60°C for 1 min. Data were analyzed by StepOne Software v2.1 (Applied Biosystems, Darmstadt, Germany). Gene expression was normalized to the expression of the reference gene *Hydroxymethylbilane Synthase* (*HMBS*) (for: 5´-CACCACAGGGGACAAGATTC-3´, rev: 5`-GTGAACAACCAGGTCCACTTC-3´).

### Western Blot Analysis

RIPA protein lysates and Western blot analysis were performed as previously described [13]. For immunoblotting, 30–60 μg of protein were used. Primary antibodies were used as follows: mouse anti-TGFBR2 (sc-17799; Santa Cruz, Dallas, USA; 1:500, 4°C, overnight); mouse anti-ß-Actin (clone 4; MP Biomedicals, Solon, USA; 1:20,000, RT, 30 min); rabbit anti-GDF-15 (HPA011191; Sigma, Taufkirchen Germany; 1:500, 4°C, overnight); rabbit anti-phospho-Smad2 and rabbit anti-Smad2 (Ser465/467) and (86F7); Cell Signaling, Danvers, USA; 1:1000, 4°C, overnight). Blots were incubated for 1 h at RT with secondary antibodies: sheep anti-mouse-IgG HRP (1:5000; GE-Healthcare, Munich, Germany) or goat anti-rabbit-IgG HRP (1:2500; Promega, Madison, USA). Detection was either performed by standard procedure using Amersham Hyperfilm ECL or ChemiDoc MP System (Bio-Rad Laboratories GmbH, Munich, Germany).

### GDF-15 Immunoprecipitation (IP)

After [^35^S]-L-methionine labeling (American Radiolabeled Chemicals, Inc., St. Louis, USA), the cell pellet was resuspended in 200 μl RIPA buffer containing 2x protease inhibitor cocktail. Protein lysates were obtained as described above. For GDF-15 immunoprecipitation, 0.3 ml of lysate containing 1.8 mg protein or 5 ml of culture supernatant were rotated with 1.5 μg GDF-15 antibody (HPA011191; Sigma, Taufkirchen, Germany) and 2x protease inhibitor cocktail overnight at 4°C. 25 μl of protein A/G agarose slurry (Oncogene Science, Uniondale, USA) were washed 3x with RIPA buffer and then incubated with the lysate or the culture supernatant and the antibody for 2 h by rotating at 4°C. Beads were washed 5x with 500 μl RIPA buffer and eluted with 2x 200 μl 1x SDS-sample buffer (106 mM Tris-HCl, 141 mM Tris Base, 2% SDS, 10% Glycerol, 0.51 mM EDTA) at 99°C for 5 min at 800 rpm. The samples were then mixed with 10 ml of scintillation cocktail and counted using a liquid scintillation analyzer (TRI-CARB 2900TR; Packard, PerkinElmer, Waltham, USA).

### Proliferation Assay

10^3^ cells were seeded in sextuplicate in 96-wells one day prior to addition of following reagents: 0.5 μg/ml Dox, 10 ng/ml human recombinant TGF-ß1 and 100 ng/ml human recombinant GDF-15. After 6 days, MTS proliferation assays was performed with the CellTiter 96 Aqueous kit (Promega, Madison, USA) according to the manufacturer‘s instruction. Spectrophotometric measurements were conducted using the GENios microplate reader (Tecan Group Ltd., Männedorf, Switzerland) and the absorbance value of the cell-free medium was substracted from the absorbance values of the samples.

## Results and Discussion

### 3.1. Identification of *de novo* synthesized TGFBR2-dependent target proteins

To ensure TGFBR2-dependent regulation, we previously generated a model cell line, herein referred to as HCT116-TGFBR2 [13]. Briefly, the HCT116 cell line is mutated for both alleles for *TGFBR2* leading to absence of the full-length protein. The two established HCT116-TGFBR2 cell lines (clone #5 and #22), however, contain a single copy integrated wildtype *TGFBR2* gene, which is expressed only in the presence of doxycycline (Dox). Time-course analysis revealed that 11-12h Dox treatment of these cells were sufficient to reach peak levels of TGFBR2 protein (**S1 Fig**).

In order to determine the TGFBR2-dependent *de novo* proteome, both HCT116-TGFBR2 clones (#5 and #22) were exposed to the ligand TGF-ß1 in the presence (TGFBR2-proficient) or absence (TGFBR2-deficient) of doxycycline and nascent proteins metabolically labeled with the methionine surrogate L-Azidohomoalanine (AHA). After click-it-mediated biotinylation, labelled proteins were captured by streptavidin-coated magnetic beads and analyzed by mass spectrometry. In order to account for proteins that show unspecific binding to the beads or proteins that reside *a priori* on the commercially available streptavidin-coated beads, the same experiment was performed in the absence of the labeling reagent (-AHA) [14]. At least 480 *de novo* synthesized proteins were detected on average in both cell clones (**S1 Table**) and under both conditions (-Dox: TGFBR2-deficient; +Dox: TGFBR2-proficient) upon stimulation with TGF-ß1 und hence remained unaffected by the TGFBR2 expression status (**Table 1**). Furthermore, cells were grown in the presence of the translation inhibitor cycloheximide (CHX) to identify bona fide *de novo* synthesized proteins (marked by asterisks in **S1 Table**). Up to 56 proteins were also identified in control cells grown in the absence of AHA, approximately 10 times less than the number of proteins identified in the presence of the AHA label, thereby confirming the specificity of this bioorthogonal labeling approach. Comparative analysis of the TGFBR2-dependent *de novo* proteome revealed 21 candidates that were found to be differentially expressed either in TGFBR2-deficient or TGFBR2-proficient cells but in at least one of three replicate samples and in each of both cell clones. Protein occurrence in both cell clones was used as the strongest selection criterion because of some variations in protein occurrence among triplicate samples. In particular, 12/21 proteins exclusively occurred in TGFBR2-deficent cells whereas the remaining proteins (9/21) were detected exclusively in TGFBR2-proficient cells that exhibit reconstituted TGF-ß signaling (**Table 2**). According to GO-term classification, differentially expressed proteins are associated with essential cellular processes like immunity (1B59), signal transduction (ASM, CSN3, DOCK8, GDF-15, TGFBR2), protein modification (CHIP, PP2AA), transcription (DTD2, RBM42, T2FA, TE2IP, TFAM, UBF1) and protein transport (ERP29, RB6I2, HGS, TMEM9). Only a few of these proteins have reportedly been linked to the TGF-ß/SMAD signaling pathway (ASM, CHIP, HGS, PP2AA, SYNE2, GDF-15) but direct regulation of their expression by the TGFBR2 receptor in colon cancer cells has not been demonstrated. Since the experimental strategy pursued in the present study focused on the identification of newly synthesized proteins, our candidate list of differentially expressed proteins most likely represent potential new *bona fide* targets of TGFBR2-mediated signaling in MSI HCT116 colorectal cancer cells.

**Table 1:** Total number of proteins identified using mass spectrometric analysis.

	**HCT116-TGFBR2 #5-AHA**			**Mean**
**-Dox**	38	26	56	40
**+Dox**	37	45	54	45
	**HCT116-TGFBR2 #5 +AHA**			
**-Dox**	583	408	656	549
**+Dox**	235	558	648	480
	**HCT116-TGFBR2 #22 +AHA**			
**-Dox**	584	541	568	564
**+Dox**	481	519	539	513

*De novo* proteins of the inducible HCT116-TGFBR2 cell lines #5 and #22 in absence (-) and presence (+) of the labeling reagent L-Azidohomoalanine (AHA) in three biological replicates in absence (-Dox) and presence (+Dox) of TGFBR2 expression and the mean value of these in triplicate identified proteins; Details on the identity of these proteins are given in S1 Table

**Table 2:** Differentially expressed and *de novo* synthesized proteins in HCT116-TGFBR2 clones #5 and #22 using mass spectrometry.

**TGFBR2-deficient**					
**Acc** ^**a**^	**Description**	**Mass [Da]**	**MASCOT Score**	**Sign. Pep.**	**Cov** ^**b**^ **[%]**
1B59	HLA class I histocompatibility antigen, B-59 alpha chain	40844	330/267	6/4	24/24
ASM	Sphingomyelin phosphodiesterase	70734	33/40	1/1	1/1
CHIP	E3 ubiquitin-protein ligase CHIP	35290	33/32/51	1/0/1	3/3/3
DTD2	Probable D-tyrosyl-tRNA(Tyr) deacylase 2	18877	47/32	0/1	11/5
ERP29	Endoplasmic reticulum resident protein 29	29032	78/48/44	1/0/0	12/10/10
HGS	Hepatocyte growth factor-regulated tyrosine kinase substrate	86708	54/43	1/1	2/1
IDHC	Isocitrate dehydrogenase [NADP] cytoplasmic	46915	100/46	2/0	9/5
RB6I2	ELKS/Rab6-interacting/CAST family member 1	128236	79/67/59	0/0/0	2/2/1
RBM42	RNA-binding protein 42	50496	39/32	1/1	4/4
T2FA	General transcription factor IIF subunit 1	58262	50/72	1/1	3/6
TE2IP	Telomeric repeat-binding factor 2-interacting protein 1	44404	44/34/43	1/1/1	2/2/2
TFAM	Transcription factor A, mitochondrial	29306	44/36/41	1/1/1	4/4/4
**TGFBR2-proficient**					
**Acc**	**Description**	**Mass [Da]**	**MASCOT Score**	**Sign. Pep.**	**Cov [%]**
CSN3	COP9 signalosome complex subunit 3	48412	54/31	1/1	4/3
DOCK8	Dedicator of cytokinesis protein 8	240886	41/50	0/1	1/1
GDF15	Growth/differentiation factor 15	34632	38/46	1/0	5/10
MYH10	Myosin-10	229827	192/46	3/0	4/1
PP2AA	Serine/threonine-protein phosphatase 2A catalytic subunit alpha isoform	36142	55/30	1/0	10/4
SYNE2	Nesprin-2	801817	44/67	0/2	0.2/0.2
TGFR2	TGF-beta receptor type-2	65951	61/34/32/80	1/1/1/1	3/2/2/3
TMEM9	Transmembrane protein 9	21074	51/34/42	1/1/1	4/4/4
UBF1	Nucleolar transcription factor 1	89692	66/42	0/0	5/4

Numbers for “score”, “peptides” and “coverage” are separated by slashes and correspond to individual experiments; Proteins listed were identified at least in one of triplicate experiments in each of both cell clones, either solely in absence of TGFBR2 (TGFBR2-deficient) or solely in TGFBR2 expressing cells (TGFBR2-proficient).

Acc, Accession; Sign. Pep., Significant Peptides; Cov, Coverage.

^**a**^ Proteins identified by the SwissProt 2013_02 database with taxonomy set to human.

^**b**^ Coverage includes all identified peptides.

Obviously, some well-known downstream targets of canonical TGF-ß/SMAD signaling like SMAD7 [15], SERPINE [16] or JUNB [17] are missing from our list of differentially expressed candidate proteins. Several reasons may account for this limitation: First, our selection criteria for candidate proteins were based on differential expression in at least one sample in each of both cell clones which might not account for variations in protein expression among these two cell line clones. For example, JUNB and CSK1 [18], another growth suppressor and TGF-ß target gene in epithelial cells, were not recruited to our list of differentially expressed candidate proteins because they were detected only in one clone of TGFBR2-proficient and TGFBR2-deficient cells, respectively. Second, our experimental strategy was designed to uncover only *de novo* synthesized proteins with clear restriction to the expression status of the TGFBR2 signal transducer and quantitative changes in protein levels upon TGFBR2 reconstitution and thus might explain why some candidates might have escaped detection. Alternatively, lack of detection might be due to differences in incorporation efficiency of methionine and its analogue AHA and/or also might depend on the number of methionine residues that actually exist in each protein. Third, TGF-ß1 ligand exposure was limited to a short time frame of 4 h that may not suffice to induce detectable levels of some newly synthesized TGF-ß1 target proteins [19]. Finally, as a caveat, we cannot exclude the possibility that some of the candidates identified in the present study actually do not represent *de novo* synthesized proteins merely because of their association to *bona fide* TGFBR2 target proteins via protein-protein interactions. Most importantly, however, we unambiguously identified the TGFBR2 protein itself in both clones of our TGFBR2-reconstituted model cell line which strongly supports the validity of our experimental approach.

### 3.2. TGFBR2-dependent expression of GDF-15

Among the *de novo* synthesized proteins specifically induced in TGFBR2-reconstituted cells, we identified the growth and differentiation factor 15 (GDF-15). Similar to TGF-ß itself, GDF-15 can exhibit divergent functions in cancer cells [20]. For example, it can act as a tumor suppressor and trigger apoptosis when overexpressed in HCT116 cells [21] but it is also found at increased levels in late stage cancers suggesting its utility as a marker of tumor progression [22]. In order to analyze TGFBR2-responsive genes in more detail, we focused on GDF-15 as one potential candidate gene because it is still unknown whether members of the TGF-ß family of cytokines can be regulated in a TGFBR2-dependent manner in colorectal cancer cells.

**Fig 1:**
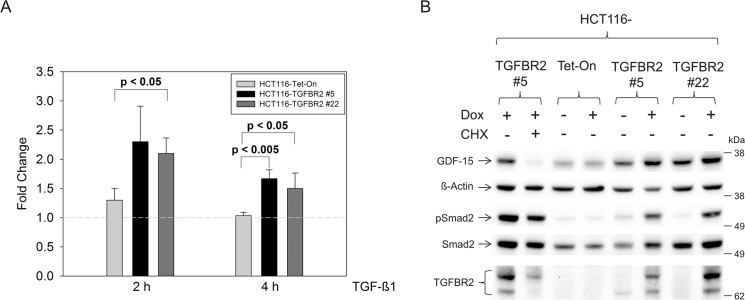
Expression analyses of GDF-15. (A) Upregulated transcript levels of GDF-15 in the presence of Dox using real-time qRT-PCR analysis. (B) Western blot analysis of GDF-15 protein expression in total protein lysates (30 μg) of TGF-ß1 stimulated cells (4 h). Expression of GDF-15 is increased when TGFBR2 is expressed (+Dox) in both cell clones but not in the parental Tet-On cell line. TGF-ß1 signaling is indicated by increased pSmad2 levels in the TGFBR2-expressing clones. Addition of cycloheximide (CHX) reduces the expression level of GDF-15. ß-Actin and Smad2 served as a loading control.

As a first step, we examined the regulation of GDF-15 expression at the transcript level. TGFBR2-proficient and TGFBR2-deficient HCT116 cells were exposed to TGF-ß1 for two different time periods (2 h and 4 h) and GDF-15 mRNA expression was determined by real-time PCR analysis (**Fig 1A**). Stimulation with TGF-ß1 for 2 h caused an approximately two-fold increase of GDF-15 transcript levels in both HCT116-TGFBR2 clones when comparing TGFBR2-proficient versus TGFBR2-deficient cells. This induction factor slightly decreased to about 1.5-fold when ligand exposure time was extended (4 h). Parental HCT116-Tet-On control cells did not show Dox-dependent alterations of GDF-15 expression which excludes any potential unspecific effects conferred by doxycycline. Apart from TGF-ß1-dependent expression changes, we also observed a difference of GDF-15 expression among both HCT116-TGFBR2 clones. In particular, fold increase of GDF-15 transcript levels was always less in clone 22 when compared to clone 5. Most likely, this might be due to the different integration sites of the *TGBFR2* transgene in these clones [13].

In addition to these transcriptional changes, we investigated whether GDF-15 protein levels are also modulated by the TGFBR2 expression status in our model system. Total cell lysates were prepared from Dox-treated and untreated HCT116-TGFBR2 clones and HCT116-Tet-On control cells after TGF-ß1 exposure (4 h) and examined by Western blot analysis (**Fig 1B**). In agreement with the transcriptional data, reconstitution of ligand-triggered TGFBR2 signaling caused an increase of GDF-15 protein levels in both cell clones whereas ß-actin levels remained unaffected. HCT116-Tet-On control cells did not show Dox-dependent changes in GDF-15 expression. Moreover, addition of cycloheximide (+CHX) decreased the expression of GDF-15 significantly in the TGFBR2-expressing cell clone #5 in contrast to ß-actin or Smad2 proteins that remained CHX resistant. These findings corroborate the *de novo* synthesis of GDF-15 and strongly support our proteomics results (**Table 2**). Increased pSmad2 levels by reconstitution of TGFBR2 (+Dox) confirms that this model system is capable to elicit proper Smad signaling.

**Fig 2:**
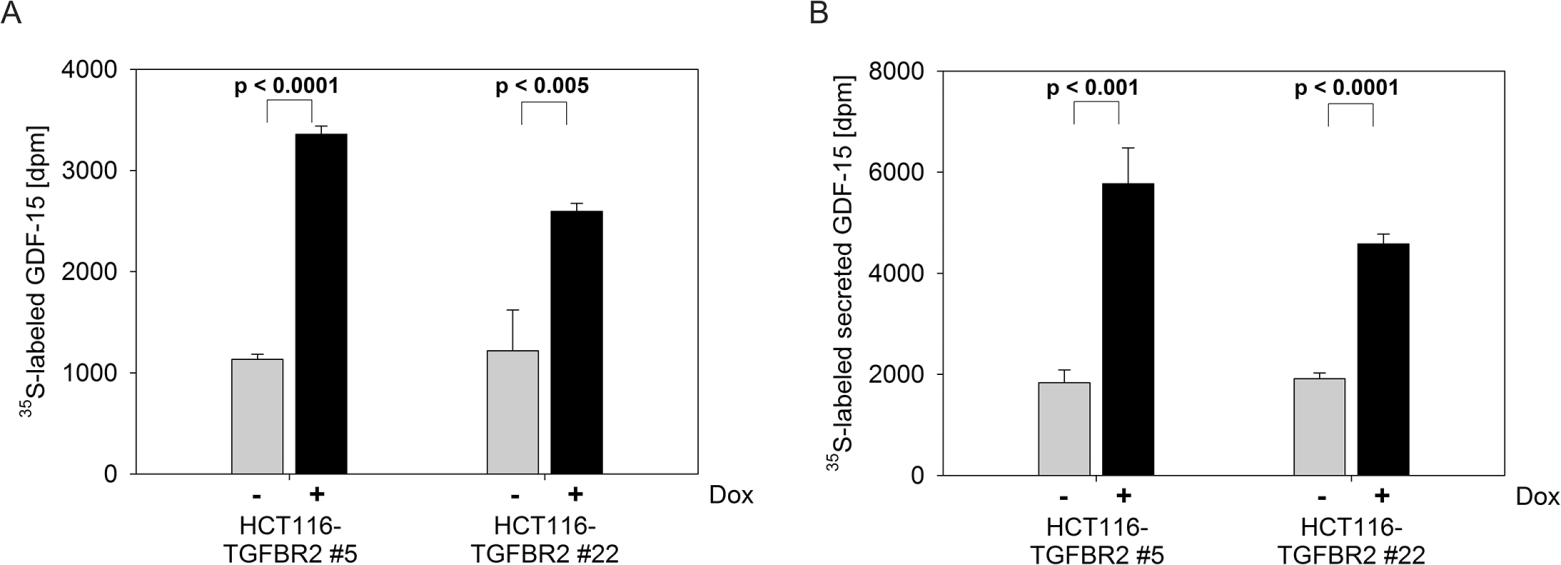
Radioactive labeling experiments. [^35^S]-L-methionine was used for metabolic labeling and subsequent immunoprecipitation of intracellular GDF-15 from total lysates (A) or secreted GDF-15 from cell culture medium (B). Cells were stimulated with TGF-ß1 for 4 h in (A) and 24 h in (B).

In order to validate the observed changes of GDF-15 protein expression in a more accurate and quantitative manner, we performed a metabolic labeling experiment using [^35^S]-L-methionine. Proteins were radioactively labeled under the same conditions as in the AHA labeling experiment but labeled GDF-15 protein was then immunoprecipitated and counted using a liquid scintillation analyzer (**Fig 2**). An approximately 2.5-3-fold increase of intracellular GDF-15 protein level was observed in both clones of Dox-treated HCT116-TGFBR2 cells upon TGF-ß stimulation compared to the basal expression level of the same cells grown in the absence of Dox (**Fig 2A**). Since GDF-15 can act in a paracrine or autocrine manner, we also analyzed the amount of [^35^S]-L-methionine labeled secreted GDF-15 protein by GDF-15 immunoprecipitation from the culture medium (**Fig 2B**). Reconstituted TGFBR2-mediated signaling in both cell clones led to a significant increase of secreted GDF-15 protein similar to the rise of intracellular levels. The parental Tet-On cell line showed no changes in newly synthesized GDF-15 expression neither in the protein lysates nor in the cell culture medium (data not shown). In conclusion, these data suggest that TGFBR2-dependent increased expression of GDF-15 seems to be conveyed to its site of action in the extracellular environment.

GDF-15 is known to exist in different forms [23]: the pro-peptide is cleaved by a protease to generate the mature bioactive protein. The mature GDF-15 protein is known to be secreted but this has also been reported for the pro-peptide [24]. The antibody used in our immunoprecipitation experiments is able to recognize the pro-peptide but does not bind to the mature form. Therefore, we would like to emphasize that our results apply to the pro-peptide version of GDF-15, which still may be attached to the mature form or may also be cleaved and independently recognized. In order to clarify whether the mature form is affected to a similar extent other antibodies need to be tested.

**Fig 3:**
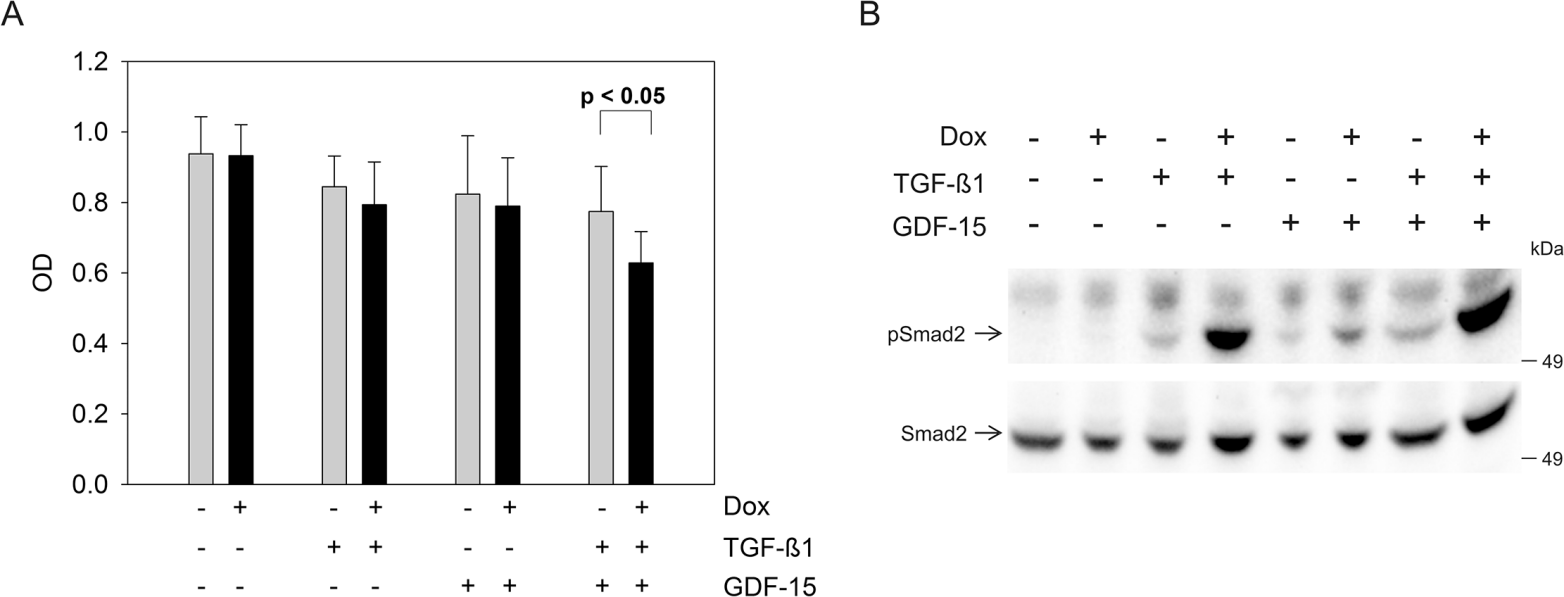
Influence of exogenous GDF-15 (100 ng/ml) on HCT116-TGFBR2 #5 cells. (A) Proliferation assay is displayed as an average of six replicates. (B) Western blot analysis of TGFBR2-dependent pSmad2 levels. HCT116-TGFBR2 #5 cells were treated in absence and presence of human recombinant TGF-ß1 (10 ng/ml) and GDF-15 for 1 h. Smad2 served as an internal loading control.

In order to test for effects of GDF-15 on cell growth and signaling, we performed a proliferation assay and pSmad2 Western blot analysis using human recombinant GDF-15 protein (**Fig 3**). TGF-ß1 or GDF-15 addition alone decreased the proliferation rate, however, the growth inhibitory effect became more prominent when both cytokines were added concomitantly when comparing TGFBR2 expressing (+Dox) to TGFBR2-deficient (-Dox) cells in the HCT116-TGFBR2 #5 (**Fig 3A**). Proliferation of the parental Tet-On cell line was not altered at all by stimulation with any cytokine and-/+ Dox in all combinations (data not shown). Furthermore, we studied the effect of exogenous addition of GDF-15 on Smad signaling. Recombinant GDF-15 alone was able to stimulate pSmad2 expression in TGFBR2 expressing cells (+Dox) but not in TGFBR2-deficient cells (-Dox) (**Fig 3B**). In the absence of recombinant GDF-15 and TGF-ß1 ligand no pSmad2 was detected, whereas stimulation with TGF-ß1 alone showed an in increase in the pSmad2 level in TGFBR2-proficient cells. TGF-ß1 and GDF-15 addition displayed the strongest increase of phosphorylated Smad2 levels which is in accordance with the observed reduced proliferation rate when both ligands were added simultaneously (**Fig 3A**). This is, to our knowledge, the first report that demonstrates phosphorylation of Smad2 by GDF-15 in a TGFBR2-dependent manner in human colorectal cancer cells. In context of cancer and disease progression where TGFBR2 is non-functional, the decrease of GDF-15 expression may lead to a growth advantage in comparison to the wildtype, TGFBR2-expressing cells, simulating rather the non-cancerous phenotype. In the Western blot analysis it is also shown that TGF-ß1 alone is increasing the level of pSmad2 expression in absence of the TGFBR2 compared to the unstimulated cells (**Fig 3B**). This basal level of pSmad2 was observed in the parental HCT116-Tet-On and the TGFBR2-expressing cell clone and is in agreement with our previous published work [13]. There is one report about potential regulation of GDF-15 mRNA by TGF-ß1 [25]. So far, this has only been observed in macrophages and there is no evidence yet about TGFBR2-dependent regulation of GDF-15 expression in other tissues or diseases like colorectal cancer. However, other studies provide some experimental evidence for the reverse situation, i.e. that GDF-15 affects TGF-ß signaling. For example, studies in mice suggest that GDF-15 can act as a potential ligand for the TGFBR2 [26]. Moreover, GDF-15 seems to modulate TGFBR2 phosphorylation or signaling as has been demonstrated in rat cerebellar granule neurons [27]. These observations do not necessarily contradict our findings but rather might indicate a potential feedback of GDF-15 on TGFBR2-mediated signaling.

Since GDF-15 has both pro- and anti-tumor activities depending on the cellular and microenvironmental context, the outcome of TGFBR2-dependent GDF-15 overexpression and increased secretion is difficult to predict. A recent study showed that circulating GDF-15 is associated with a higher risk of CRC and that nonsteroidal anti-inflammatory drugs (NSAIDs) may lower the risk [28]. Controversially, there are publications showing that these NSAIDs induce expression of GDF-15 which then leads to apoptosis in HCT116 cells [21, 29], which is in agreement with the observations we made in this study: TGFBR2 reconstitution represents rather the wildtype situation in which GDF-15 is expressed at higher levels, whereas in the tumor state (TGFBR2 deficiency) less GDF-15 is expressed and secreted and the tumor cells may escape apoptosis.

In terms of the type II receptors that transduce the signals elicited by the superfamily of TGF-ß/Bone morphogenic protein (BMP)/Activin ligands, proteomics data derived from TGF-ß1 stimulated and TGFBR2-reconstituted cells in the present study enable a direct comparison with proteomics data gained from our previous work on activin A-stimulated and ACVR2-reconstituted cells. Compared to the *de novo* proteome of HCT116-ACVR2 cells [14], there is no overlap with the differentially expressed set of candidate genes identified in HCT116-TGFBR2 cells. Although both receptors are members of the same family of type II signal transducers and transmit their signals through common SMAD mediator proteins in the canonical pathway, the observed lack of overlap might in part be due to binding to different ligands.

Overall, using a well-characterized model system that allows inducible expression of a major driver of colon tumorigenesis in an isogenic background, we identified a set of *de novo* synthesized candidate proteins whose differential expression appears to be regulated in a TGFBR2-dependent manner. Among these candidates we identified the TGF-ß superfamily member GDF-15 as a potential novel target whose mRNA, intracellular and secreted protein levels were upregulated upon TGFBR2 reconstitution. This TGFBR2-GDF15 link might be one part of a regulatory loop that also involves a previously described GDF-15-TGFBR2 link [27]. Our methodical approach opens a new field of investigations to untangle the many unknown effects of TGFBR2 signaling not only in MSI CRC, but also in other diseases with functionally impaired TGF-ß signaling.

## Supporting Information

S1 Fig Western blot analysis of doxycycline (Dox)-dependent TGFBR2 expression. Time course experiment of TGFBR2 expression in HCT116-TGFBR2 #5 with Dox for 3 h, 6 h, 12 h and 24 h in duplicate and without Dox (-). ß-Actin served as a loading control.
(TIF)

S1 Table List of proteins identified by mass spectrometry analysis in presence of the labeling reagent L-Azidohomoalanine (AHA). The click-it approach was applied prior to mass spectrometry to allow the identification of newly synthesized proteins. The data were obtained for both cell clones, HCT116-TGFBR2 #5 and #22, in triplicate in presence and absence of TGFBR2 expression. Asterisks indicate proteins that were identified in presence of cycloheximide (CHX), an inhibitor of protein biosynthesis and hence are not newly synthesized.
(XLSX)
