# Supplementary figures and images for: Reconstitution of TGFBR2-Mediated Signaling Causes Upregulation of GDF-15 in HCT116 Colorectal Cancer Cells
Source: PLoS One. 2015 Jun 26;10(6):e0131506. doi: 10.1371/journal.pone.0131506 (PMC4484253; doi:10.1371/journal.pone.0131506)

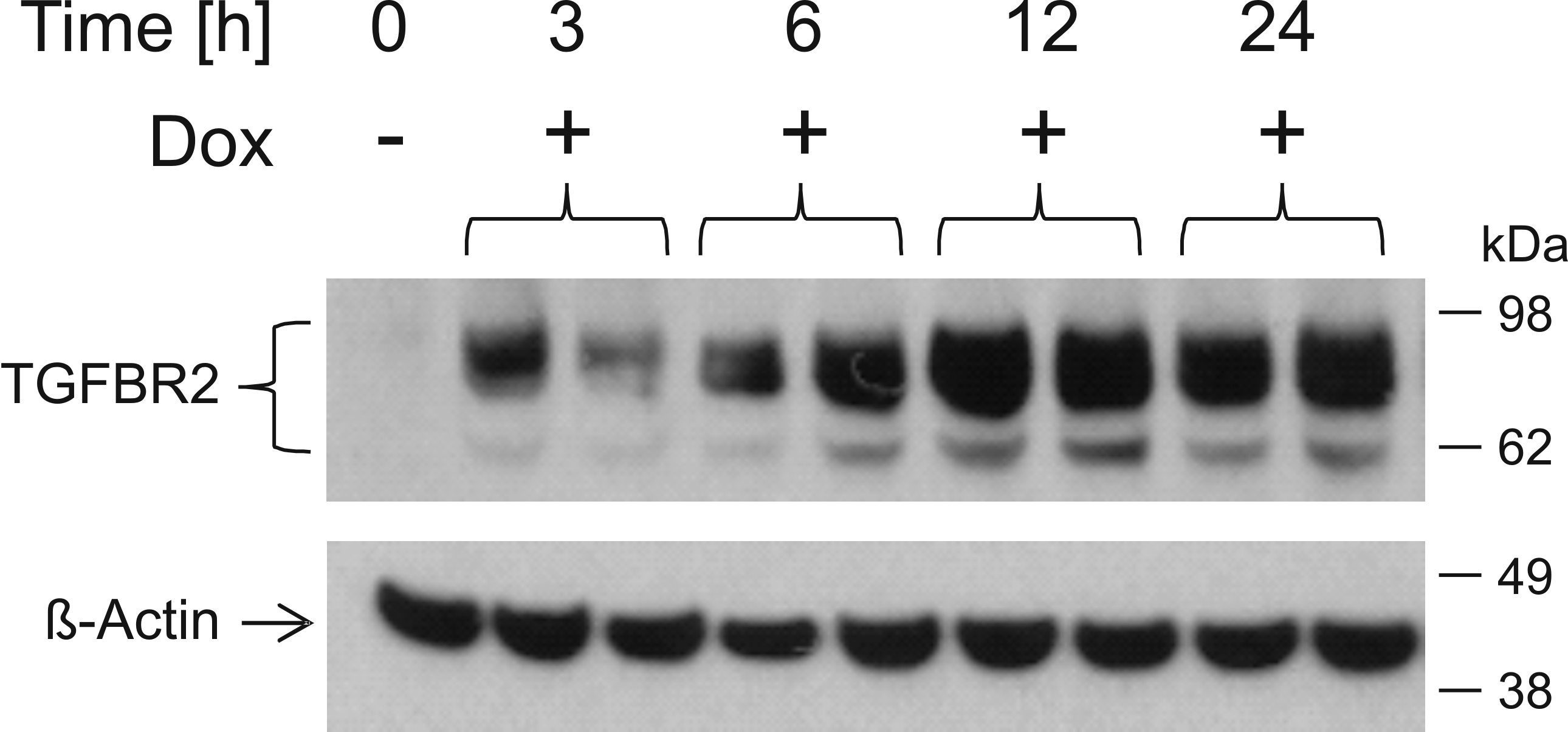

Supplement: S1 Fig — Time course experiment of TGFBR2 expression in HCT116-TGFBR2 #5 with Dox for 3 h, 6 h, 12 h and 24 h in duplicate and without Dox (-). ß-Actin served as a loading control. (TIF) [file pone.0131506.s001.tif]
